# Why is the European Unifying Recommendation for Treating Syndrome of Inappropriate Antidiuretic Hormone (SIADH) and Cerebral Renal Salt Wasting Not Commonly Recommended in the US

**DOI:** 10.7759/cureus.100943

**Published:** 2026-01-06

**Authors:** Louis Imbriano

**Affiliations:** 1 Division of Nephrology and Hypertension, New York University (NYU) Langone Hospital, Mineola, USA

**Keywords:** cerebral renal salt wasting, hyponatremia, inflammation, siadh, treatment

## Abstract

Hyponatremia in patients with the syndrome of inappropriate antidiuretic hormone (SIADH) and in patients with cerebral renal salt wasting (CRSW) requires radically different treatment. Delay in proper treatment often occurs because the laboratory presentation of both syndromes is identical, and the fact that CRSW is considered very rare. The parameters observed are hyponatremia, hypouricemia with high fractional excretion of uric acid, normal renal, adrenal and thyroid function, concentrated urine with urine osmolality higher than plasma osmolality, and urine sodium concentration often > 30 mmol/L. The treatment of SIADH requires fluid restriction or forced fluid excretion, while the treatment of CRSW requires fluid administration. Delays in treatment may result in serious, possibly fatal complications. A unifying treatment protocol has been developed in Europe but is often ignored in the American literature. The European approach introduces a safer treatment of severe hyponatremia when the physician is confronted with either SIADH or CRSW. It recommends initial treatment with boluses of 3% saline over 20 minutes via a peripheral vein, with modifications of the treatment to increase serum sodium by 5 mEq. over the next 1-2 hours.

## Editorial

The original description of the syndrome of inappropriate antidiuretic hormone (SIADH) referred to ectopic secretion of ADH by a tumor. However, in the decades since then, there have been thousands of so-called SIADH reports describing hyponatremia in many patients with diverse and unrelated disorders other than a neoplasm. This is likely due to the under-recognition that inflammation induces inappropriate ADH secretion. This editorial is intended to show that patients with SIADH, whether caused by a tumor or by inflammation, have inappropriate levels of antidiuretic hormone (ADH), while patients with cerebral renal salt wasting (CRSW) have appropriate levels of ADH. Because the pathophysiology of the two syndromes is completely different, they must be treated differently. The water-overloaded SIADH patient should be water-restricted or undergo a forced water diuresis, whereas the dehydrated CRSW patient should have solute administration. Unfortunately, physicians are unable to assess patients’ volume status at the bedside due to the lack of immediate methods to accurately assess blood volume. These methods may include measuring serum aldosterone, norepinephrine, bioelectrical impedance, central venous pressure (CVP), pulmonary capillary wedge pressure (PCWP), transpulmonary thermodilution, as well as physical exam. Some of these measures are invasive or unavailable at the bedside and delay critical treatment of hyponatremia. 

The vast literature regarding the treatment of SIADH suggests that treatment should be based on total body water, effective circulating volume, or whether the patient is hypervolemic or euvolemic. However, this is not possible at the bedside when the patient needs urgent care [1]. Unfortunately, CRSW has been regarded as a “controversial issue”, or “rare”, being described in textbooks in a few lines, or “ghosted” in present-day parlance. This has led to widespread disinterest in its prevalence and to the avoidance of best practices. CRSW is more common than expected and has been identified in patients with pneumonia, hip fracture, Herpes Zoster, B-cell lymphoma, cellulitis, Lyme disease and urinary tract infection. . A study of 62 hospitalized patients with hyponatremia revealed a remarkable and unexpected frequency of CRSW in 38% of the patients studied [2]. This study was a single-center study which has not been replicated and remains controversial at this time. Recently, especially in neurosurgical units, there has been broad acceptance of CRSW but silence in the world of internal medicine. Calling CRSW “rare” raises the specter of an ancient Latin proverb: “Qui tacet consentire videtur” or “He who is silent seems to consent”.

Hyponatremia is the most common electrolyte disorder in hospitalized patients. The causes are numerous, and in many cases, the patient’s history, physical exam, medication history, urine protein excretion, hormone assessment (thyroid, cortisol, aldosterone), plasma lipids and proteins, as well as plasma osmolality, will reveal the etiology. Once these causes are eliminated, the nephrologist is left to treat SIADH or CRSW.

The first report of SIADH involved inappropriate secretion of ADH by a lung tumor [3]. But there are dozens of other causes of SIADH that are not due to ADH secretion by a tumor and occur in very diverse groups of disorders, which, until the beginning of the 21st century, seemed to have no common pathogenesis. Inflammation is now recognized as the cause of inappropriate ADH secretion.

The differential diagnosis of hyponatremia is extraordinarily broad and includes (a) Primary polydipsia and Beer Potomania: (ingestion of large amounts of hypotonic fluids), (b) Hypertonic: (due to plasma content of glucose, mannitol, glycine, maltose), (c) Spurious: (due to high plasma levels of proteins or lipids), (d) Nephrogenic SIADH: (activating mutation of the AVP receptor-2 in the collecting ducts), (e) Hypothyroidism: (likely due to decreased cardiac output, reduced “effective blood volume” sensed by carotid and aortic baroreceptors, and appropriately increased ADH), (f) Adrenal Insufficiency: (due to lack of cortisol’s tonic inhibition of ADH, as well as aldosterone deficiency leading to urinary sodium wasting, volume depletion and appropriate stimulation of ADH secretion), (g) Reset Osmostat: (several conditions can lower the normal plasma osmolality threshold), (h) SIADH: caused by medication-induced secretion of ADH (especially NSAIDS, thiazide diuretics, SSRIs, antipsychotics, antiepileptics, desmopressin, tricyclic antidepressants, phenothiazines, cyclophosphamide, vincristine, either by increasing secretion of ADH or potentiating its renal activity. Or initiated by the inappropriate release of ADH by tumors, pulmonary diseases, infections, CNS diseases, possibly induced by the cytokine, interleukin (IL)-6, which directly induces ADH secretion, (i) CRSW: a proximal tubule natriuretic factor causes sodium wasting (as well as urate wasting), which results in extracellular fluid volume depletion and an appropriate induction of ADH (possibly due to haptoglobin-related protein without signal peptide (HPRwoSP)). The mechanisms mentioned above (points a to i) may not represent a complete physiological explanation for the development of hyponatremia.

SIADH can be related to medications that directly induce ADH secretion from the pituitary gland or potentiate the effects of ADH action in the kidney. But for many other seemingly unrelated conditions, such as head trauma [4], or sub-arachnoid hemorrhage [5], pain [6], stress [7], infections [8], and autoimmune disorders [9], the unifying stimulus for ADH secretion is the direct action of the inflammatory cytokine IL-6 on the hypothalamus. These forms of inflammation-induced SIADH are also due to inappropriate ADH secretion and are often included in lists that include the classic SIADH cases.

Quite the contrary, hyponatremia in patients with CRSW is due to appropriate ADH secretion. Recently, Maesaka et al. identified a potential biomarker, HPRwoSP, an acute phase reactant, which likely causes CRSW [10]. In rat studies, HRPwoSP is a natriuretic peptide that inhibits proximal tubule reabsorption of sodium and uric acid. It is presumed that genetically predisposed patients with some of the same conditions mentioned above generate HPRwoSP and present with CRSW, while others respond to IL-6 and present with SIADH. 

CRSW patients become progressively volume-depleted, which activates central baroreceptors to stimulate the appropriate secretion of ADH, with increased reabsorption of water in the distal tubules, resulting in dilutional hyponatremia. Further complicating the differential diagnosis is the variable urinary sodium level if the patient has had recent diuretic use, renal insufficiency, heart failure, hypotension, or restricted oral solute intake. Hence, both SIADH and CRSW create a treatment dilemma, vexing physicians. Severe hyponatremia associated with seizures, coma, or altered mental status mandates immediate treatment, which may require fluid restriction if the patient has SIADH or fluid administration if the patient has CRSW.

Western literature regarding the treatment of SIADH is correct. There are multiple algorithms and charts which include water restriction [11], hypertonic saline [12,13], Desmopressin [14], Declomycin [15], urea [16], urea transporter inhibitors [17,18], loop diuretics [19], vasopressin receptor antagonists (the “vaptans”) [20,21], and sodium-glucose transporter inhibitors (SGLT2i) [22]. The use of urea transporter inhibitors or SGLT2i’s are currently being evaluated for safety and would only be safe if the patient was accurately diagnosed with SIADH. All articles stress that the rate of sodium correction is crucial to avoid central pontine myelinolysis. Despite the plethora of the above-mentioned treatments, which are useful for the “wet” patient with SIADH, they can be catastrophic for the volume-depleted patient with CRSW. Sadly, the treatment of patients with CRSW is poorly advanced and barely mentioned in American literature. 

The danger arises when the urgency of treatment diverts the physician to volume-depleting or water-restricting the patient with “SIADH”, assuming the so-called rarity of CRSW. Fluid restriction or induced water excretion in a patient who actually has CRSW can result in shock, organ failure, or even death. Even a slight increase in serum sodium with 3% saline can improve symptoms and reduce the immediate risk of severe neurological injury [23]. Some investigators have suggested that treatment of hyponatremia in these two diverse scenarios should avoid fluid restriction or diuresis [24].

The European Society of Endocrinology (ESE), the European Renal Association-European Dialysis and Transplant Association (ERA-EDTA), and the European Renal Best Practice group (ERBP) subtly acknowledge the danger of erroneous treatment and published a universal, standard, and all-embracing guideline which avoids deciding whether a patient has SIADH or CRSW [25]. Their recommendations advise initial therapy of all patients with hyponatremia, whether SIADH or CRSW, and exhibiting severe symptoms such as seizures, coma, or altered mental status should commence with 150 ml 3% saline over 20 minutes (via a peripheral vein), repeating serum sodium after 20 minutes and repeating another 150 ml 3% saline, if necessary, until a target of 5 mEq/L increase in serum sodium is achieved within one to two hours. The immediate goal is to increase serum sodium by 4-6 mEq/L within four to six hours, and certainly no more than 8 mEq./L within 24 hours.

Once a slight improvement is achieved, fluid administration should be continued with 0.9% normal saline or half-normal saline, checking serum sodium every four to six hours to ensure that sodium correction will not exceed 8 mEq/L during the first 24 hours to minimize or prevent osmotic demyelination syndrome. Using this approach is “etiology-diagnostic”. It may avoid serious and fatal complications of hyponatremia and allows time for further evaluation and monitoring the trends of serum sodium after initial correction. After partial correction of hyponatremia all attempts should be made to determine volume status and clarify whether the patient has SIADH or CRSW. And to limit correction to only 8 mEq/L per day for subsequent days until serum sodium reaches 130 mEq/L. It is worth noting that correcting any coexisting hypokalemia will accelerate the correction of serum sodium. Employing this universal treatment guideline will prevent the error of mistaking SIADH (with its attendant “water restriction/deprivation” recommendation) for CRSW (which requires volume replacement). It allows for timely, safer on-site management of the hyponatremic patient, preventing further deterioration. Hopefully, this European unifying protocol for treating "SIADH" and CRSW will be highlighted in the American medical community to promote broader acceptance.
